# Prognostic effect of activated EGFR expression in human colon carcinomas: comparison with EGFR status

**DOI:** 10.1038/sj.bjc.6605473

**Published:** 2009-12-08

**Authors:** R L Rego, N R Foster, T C Smyrk, M Le, M J O'Connell, D J Sargent, H Windschitl, F A Sinicrope

**Affiliations:** 1 Division of Oncology, Mayo Clinic, Rochester, MN 55905, USA; 2 Division of Gastroenterology and Hepatology, and Miles and Shirley Fiterman Center for Digestive Diseases, Mayo Clinic, Rochester, MN 55905, USA; 3 Biomedical Statistics and Informatics, Rochester, MN 55905, USA; 4 Pathology and Loboratory Medicine, Mayo Clinic, Rochester, MN 55905, USA

**Keywords:** EGFR, phosphorylated EGFR, prognosis, colon cancer, proliferation, apoptosis

## Abstract

**Background:**

Evidence suggests that epidermal growth factor receptor (EGFR)-activation status may better predict the clinical behaviour of colon cancers than does EGFR expression. However, the prognostic effect of phospho-EGFR in primary colon cancer remains undefined.

**Methods:**

Phospho-EGFR (Tyr-1173) and EGFR expression were analysed by immunohistochemistry (IHC) in tissue microarrays of TNM stage II and III colon cancers from completed adjuvant therapy trials (*n*=388). Staining intensity was scored and correlated with clinicopathological variables, DNA mismatch repair (MMR) status, rates of cell proliferation (Ki-67), apoptosis (caspase-3), and patient survival.

**Results:**

Phospho-EGFR expression was detected in 157 of 388 (40%) tumours, whereas EGFR was found in 214 of 361 (59%). Although phospho-EGFR was unrelated to clinicopathological variables, strong EGFR intensity was associated with higher tumour stage (*P*=0.03). Tumours overexpressing EGFR (*P*=0.0002) or phospho-EGFR (*P*=0.015) showed increased Ki-67, but not caspase-3 expression. Phospho-EGFR was not prognostic. EGFR intensity was associated with worse disease-free survival (DFS) (hazard ratio (HR): 1.21 (1.03, 1.41); *P*=0.019) and overall survival (OS) (HR: 1.19 (1.02, 1.39); *P*=0.028). Tumours expressing both EGFR and phospho-EGFR had similar survival as EGFR alone. Stage and lymph node number were prognostic for DFS and OS, and histological grade for OS. EGFR was an independent predictor of DFS (*P*=0.042) after adjustment for stage, histological grade, age, and MMR status.

**Conclusion:**

Phospho-EGFR and EGFR expression were associated with tumour cell hyperproliferation. Phospho-EGFR was not prognostic, whereas increased EGFR intensity was independently associated with poor DFS.

The epidermal growth factor receptor (EGFR) is a transmembrane glycoprotein and receptor tyrosine kinase (TK) that is encoded by the *c-erbB-1* proto-oncogene (Yarden and Sliwkowski, 2001). EGFR signalling is activated by ligands that induce homo- or heterodimerisation with other ErbB family members (Yarden and Sliwkowski, 2001). Binding of a ligand to the extracellular domain of EGFR results in receptor dimerisation and autophosphorylation on tyrosine residues with the major phosphorylation site being tyrosine 1173 (Tyr-1173) (Lombardo *et al*, 1995; Chattopadhyay *et al*, 1999). EGFR activation triggers intracellular signalling that results in increased proliferation and a number of processes related to cell survival (Yarden and Sliwkowski, 2001; Buettner *et al*, 2002). EGFR expression is implicated in the pathogenesis of colorectal cancer (Roberts *et al*, 2002) and a direct relationship between EGFR expression by colon cancer cells and their ability to produce hepatic metastasis has been shown in preclinical models (Radinsky *et al*, 1995).

Evidence suggests that EGFR-activation status may be more important than receptor expression (Piazzi *et al*, 2006). However, only limited data exist for phospho-EGFR in human colorectal cancers and highly variable expression frequencies were reported using an anti-Tyr 1068 antibody (Cunningham *et al*, 2005; Personeni *et al*, 2005). Till date, the prognostic effect of phospho-EGFR expression in human colon cancers has not been addressed. A polymorphism in the *EGFR* gene (R497K) was associated with a marked decrease in EGFR phosphorylation and was reported to be a favourable prognostic marker in stage II/III colorectal cancers (Wang *et al*, 2007). In contrast to phospho-EGFR, EGFR expression in primary colon cancers has been shown to be an adverse prognostic marker in some (Lee *et al*, 2002; Resnick *et al*, 2004), but not other (McKay *et al*, 2002; Spano *et al*, 2005), studies and an evaluation of its prognostic effect using the FDA-approved antibody and methodology is lacking.

Recently, elongation of a microsatellite repeat at the 5′ untranslated region (UTR) was found in colon cancers with microsatellite instability (MSI), but not in microsatellite stable tumours (Baranovskaya *et al*, 2009). Elongation of this repeat was shown to result in the downregulation of EGFR mRNA levels in human colon cancer cells and in human tumours with MSI. This report, however, did not examine EGFR protein expression levels in relation to MSI status. MSI results from defective DNA mismatch repair (MMR) and these tumours show distinct phenotypic features and are reported to have better stage-adjusted survival rates compared with tumours with intact MMR (Sinicrope and Sargent, 2009).

In this report, we determined the prognostic effect of EGFR expression in relation to its activation status in primary stage II and III colon carcinomas from patients treated in 5-fluorouracil-based adjuvant therapy trials. We utilised antibodies against the major EGFR phosphorylation site Tyr-1173 and utilised the EGFR pharmDx kit. The association of EGFR with DNA MMR status, and phospho-EGFR or EGFR expression with cell proliferation and/or apoptosis were also studied.

## Materials and methods

### Patients and specimens

Surgically resected, primary colon carcinomas were analysed from patients who participated in randomized 5-fluorouracil-based adjuvant chemotherapy trials conducted by the North Central Cancer Treatment Group (NCCTG: 91-46-53, 79-46-04, 89-46-51). Details of these studies have been previously reported (Allegra *et al*, 2002; O'Connell *et al*, 2006). Paraffin-embedded tumour blocks were available from a non-random patient subset of the overall study populations. Phospho-EGFR was analysed in 388 patients with TNM stage II and III colon carcinomas. EGFR was studied in 303 stage II and III cases from a single adjuvant trial (91-46-53). Tumour histological grade was categorized as: grade 1, well differentiated; grade 2, moderately differentiated; grade 3, poorly differentiated; and grade 4, undifferentiated (Compton *et al*, 2000).

All patients were censored at 5 years after randomisation for disease-free survival (DFS) and were followed for a median of 8 years for overall survival (OS).

### Tissue microarrays (TMA)

Tissue microarrays had been constructed from paraffin tumour blocks (Beecher Instruments, Silver Spring, MD, USA). Each patient had three tumour cores and two normal cores consisting of normal liver, tonsil or placenta that were used as Immunohistochemistry (IHC) controls and navigation markers (Jourdan *et al*, 2003). Tissue sections (4–6 *μ*m) were cut from TMA blocks for IHC.

### Immunohistochemistry

Slides were deparaffinized, endogenous peroxidase activity was blocked and staining was carried out using a DAKO Cytomation Autostainer (DAKO, Carpinteria, CA, USA). For phospho-EGFR, heat-induced epitope retrieval was carried out in EDTA using a steamer (30 min, 98–100°C). Slides were placed into a wash buffer (Tris-buffered saline solution containing 0.05% Tween 20, pH 7.6 (DAKO)) and then a serum-free protein block was applied (DAKO) for 10 min. After rinsing in wash buffer, slides were incubated overnight at 4°C with the anti-phospho-EGFR (Tyr-1173) mouse monoclonal antibody (DAKO), diluted 1 : 100. After rinsing, a secondary antibody system (advance HRP (DAKO)) was applied for 15 min. For EGFR, non-specific antibody binding was inhibited using a protein block BACKGROUND*SNIPER* (Biocare Medical, Walnut Creek, CA, USA). Slides were stained for EGFR using the Dako pharmDx kit (DAKO) as per the manufacturer's instructions. Colour was developed using diaminobenzidine followed by counterstaining with hematoxylin. For both antibodies, a breast carcinoma was used as a positive control and a negative control omitted the primary antibody but included all other steps.

For the analysis of Ki-67 and caspase-3, antigen retrieval was carried out using a heat-inactivated epitope retrieval system, EDTA*DECLOAKER* (Biocare). Double staining for caspase-3 and Ki-67 was carried out by incubating sections in a prediluted monoclonal and polyclonal antibody double stain cocktail, Ki-67(M)+caspase-3(R) (Biocare) for 60 min. Slides were then incubated with MACH 2 (HRP(ms)+ALP(rb)) secondary antibody cocktail (Biocare) for 30 min. Next, sections were incubated in Cardassian DAB (HRP) to visualise Ki-67 and in Vulcan Fast Red (ALP) to visualise caspase-3 (both reagents from Biocare). Slides were counterstained in hematoxylin.

### IHC scoring

Phospho-EGFR was scored as having either weak/moderate (1+ and 2+) or absent staining intensity in the tumour cytoplasm. Membranous EGFR staining was scored as per the manufacturer's instructions (Guidelines for Interpreting EGFR pharmDx, DAKO), and intensity was categorised as follows: 0 (no staining), 1+ (incomplete circumferential staining), 2+ (complete circumferential staining), and 3+ (complete strong circumferential staining). We regarded tumours with >5% of stained tumour cells to be positive for either marker. Staining results were found to be very similar among cores and in the event of a difference, we selected the core with higher staining intensity provided that that tumour–to–background signal was optimal. Staining criteria were reviewed in a random subset by two pathologists (RLR, TCS) before scoring all cases. All specimens were then analysed by a pathologist (RLR) without the knowledge of clinical information.

Ki-67 and caspase-3 staining were scored using computerised TMA images of individual tissue core images on a given slide (Slide Scanner; Bacus Laboratories, Lombard, IL, USA), as previously described (Sinicrope *et al*, 2007). The number of Ki-67- and caspase-3-positive cells was determined, and mean and median values were calculated. The caspase-3 : Ki-67 ratio was determined by dividing number of caspase-3 events by number of positive Ki-67 cells.

### DNA MMR status

Defective DNA MMR was defined as absent expression of an MMR protein by IHC and instability at the BAT 26 locus, as previously described (Thibodeau *et al*, 1998; Parc *et al*, 2000; Garrity *et al*, 2004) for adjuvant study 91-46-53. For the other studies, MSI was analysed as previously described (Thibodeau *et al*, 1998; Halling *et al*, 1999) and tumours with high frequency instability (MSI-H, ⩾30% of the loci demonstrating MSI) (Boland *et al*, 1998; Halling *et al*, 1999) were regarded as showing defective MMR, whereas the others were categorized as showing intact MMR.

### Statistical analysis

Chi-square tests were used to test for an association between categorical variables and the Wilcoxon rank-sum test was used to test for an association between a dichotomized variable and a continuous variable. OS (censored at 8 years) was calculated as the number of years from random assignment to the date of death or last contact. DFS (censored at 5 years) was calculated as the number of years from random assignment to the date of disease recurrence or death. The distributions of OS and DFS were estimated using Kaplan–Meier methodology. Univariate and multivariate Cox's proportional hazards models (Cox, 1972) were used to explore the association of clinical and laboratory parameters with OS and DFS. Graphical methods were used to examine whether the underlying model assumptions were satisfied (e.g., proportional hazards) (Grambsch and Terry, 1994). Statistical tests were two sided, with *P*⩽0.05 considered significant. *P*-values were not adjusted for multiple comparisons. Statistical analyses were carried out using SAS software (SAS Institute, Cary, NC, USA).

## Results

### Study population

Patient demographic data and clinicopathological features of the colon carcinomas examined in this study are shown in Table 1. Patients whose tumours were analysed for phospho-EGFR had a median age of 64 years (mean 62.5, range 26–85). For EGPR, median age was 64 years (mean 62, rage 30–83).

### Phospho-EGFR, EGFR expression, and clinicopathological features

**Figure 1:**
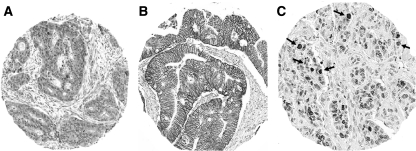
Immunohistochemical analysis of phospho-epidermal growth factor receptor (EGFR), EGFR, Ki-67, and caspase-3. (**A**) Colon carcinoma shows phospho-EGFR staining in cytoplasm of tumour cells. Magnification: × 10. (**B**) Colon carcinoma shows membranous EGFR staining intensity (2+). Magnification: × 10. (**C**) Dual staining for caspase-3 (*red*) and Ki-67 (*brown*) allows apoptotic cells to be analysed in context of proliferating cells. Magnification: × 20. The color reproduction of this figure is available on the html full version of the manuscript.

Phospho-EGFR (Tyr-1173) expression was detected in 157 of 388 (40%) colon cancers (Table 1). Staining intensity was weak/moderate (1+ and 2+) and localised to the tumour cytoplasm (Figure 1A). EGFR expression was analysed using the EGFR pharmDx kit that was approved by the United States Food and Drug Administration to assess patient eligibility for treatment with anti-EGFR antibodies. We found that 214 of 361 (59%) colon carcinomas examined showed EGFR plasma membrane staining (1–3+). Absent/weak/moderate EGFR intensity (0, 1+, and 2+) was detected in 301 (83%) tumours and complete strong circumferential staining (3+) was found in 60 (17%) (Table 1, Figure 1B).

Phospho-EGFR expression did not correlate with the clinicopathological variables (Table 2). Colon cancers with strong EGFR (3+) *vs* other intensities (0–2+) were significantly more likely to be stage III (87 *vs* 73%; *P*=0.03) (Table 2). EGFR intensity was unrelated to patient age, gender, or tumour site (Table 2). We also compared mean and median EGFR values with the clinicopathological variables. Poor/undifferentiated tumours had significantly higher EGFR intensity compared with tumours with well/moderate histological grade (*P*=0.007) (data not shown). Expression of EGFR and phospho-EGFR were not correlated in a patient subset wherein both proteins were analysed (*n*=190; *P*=0.45). However, 32 of the 190 cases expressed phospho-EGFR but were negative for EGFR. When these 32 cases were excluded, EGFR expression was significantly correlated with phospho-EGFR (*P*<0.0001).

We found that 33 of 360 (9%) colon cancers showed defective DNA MMR and 327 (91%) showed intact MMR (Table 1). Tumours with defective MMR were significantly more likely to show proximal location, poor/undifferentiated histology, and to be from older aged patients compared to cases with intact MMR (data not shown), as previously reported (Lothe *et al*, 1993; Thibodeau *et al*, 1993, 1998; Halling *et al*, 1999; Gryfe *et al*, 2000; Sinicrope *et al*, 2006a, 2006b). Recently, elongation of a microsatellite repeat at the 5′ UTR was found in colon cancers with defective MMR, but not in those with intact MMR (Baranovskaya *et al*, 2009), and elongation of this repeat was associated with downregulation of EGFR mRNA levels. Accordingly, we compared EGFR expression with MMR status in our study population. As a dichotomised variable, EGFR intensity was unrelated to MMR status (Table 2). Mean and median EGFR intensity were, however, increased in tumours with defective *vs* intact MMR, but did not reach statistical significance (*P*=0.065).

### Association of phospho-EGFR and EGFR with cell proliferation and apoptosis

Tumour growth rates are governed by the counterbalancing processes of cell proliferation and apoptosis, as measured here by Ki-67 and caspase-3 expression in a patient subset (*n*=190) (Figure 1C). Weak/moderate (1+ and 2+) staining intensity (*vs* absent) for phospho-EGFR was associated with a higher mean and median number of Ki-67-positive tumour cells (*P*=0.015) (Table 2). Furthermore, tumours with strong (3+) EGFR staining intensity (*vs* other) had a higher mean and median number of Ki-67-positive tumour cells (*n*=361; *P*=0.0002) (Table 2). Tumours with poor/undifferentiated histology had a higher mean and median number of Ki-67-positive tumour cells as compared with tumours with well/moderate histologic grade (*P*=0.0004) (data not shown). Phospho-EGFR staining and strong EGFR staining intensity were not significantly associated with caspase-3 expression nor with the caspase-3/ki-67 ratio (Table 2).

### Association of phospho-EGFR and EGFR with patient survival

**Figure 2:**
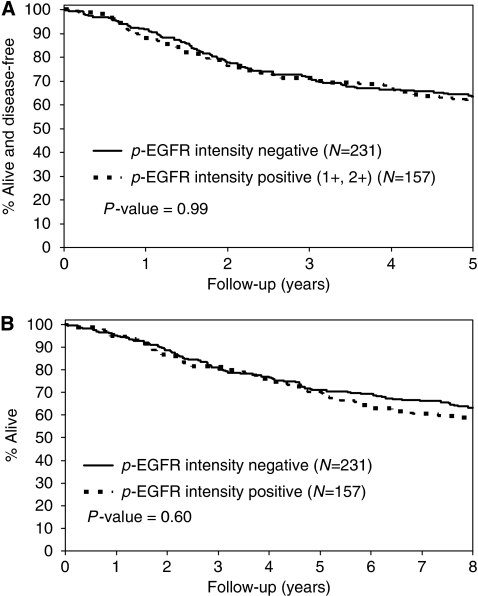
Univariate analysis of phospho-epidermal growth factor receptor (EGFR) staining intensity and (**A**) disease-free survival (DFS) for p-EGFR positive *vs* negative and (**B**) overall survival (OS) rates for p-EGFR positive *vs* negative in stage II and III colon carcinomas (*n*=388) from patients treated in adjuvant therapy trials.

**Figure 3:**
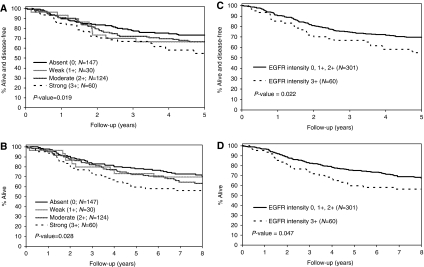
Univariate analysis of continuous epidermal growth factor receptor (EGFR) staining intensity showing all the four categories and continuous EGFR *P*-value for (**A**) disease-free (DFS) and (**B**) overall survival (OS) rates in patients with stage II and III colon carcinomas (*n*=361) treated in adjuvant therapy trials. The prognostic effect of EGFR intensity analysed as a dichotomised variable is also shown for (**C**) DFS and (**D**) OS in these patients (*n*=361).

The median duration of follow-up for patients who remain alive was 8 years. We analysed phospho-EGFR and EGFR staining intensity as continuous variables. In a univariate analysis, patient tumours expressing phospho-EGFR had similar DFS and OS survival rates (Figure 2) as did tumours lacking phospho-EGFR (Table 1). Increasing EGFR intensity was associated with significantly shorter 5-year DFS (hazard ratio (HR) (95% CI): 1.21 (1.03, 1.41), *P*=0.019) and OS (HR: 1.19 (1.02, 1.39), *P*=0.028) rates (Table 1; Figure 3A and B). We also analysed EGFR as a dichotomised variable and compared tumours with absent/weak/moderate EGFR intensity (0, 1+, and 2+) *vs* strong (3+) staining (Figure 3C and D). We found that patients with tumours that showed strong staining had significantly reduced 5-year DFS (HR (95% CI): 1.65 (1.07, 2.53), *P*=0.022) and OS (HR: 1.55 (1.00, 2.39), *P*=0.47) rates (Table 1). We then determined whether tumours expressing both EGFR and phospho-EGFR (*n*=158) had a poorer prognosis compared with those with EGFR alone. However, no differences in survival were found indicating that activation of EGFR Tyr-1173 does not further affect prognosis in EGFR-expressing tumours. Neither Ki-67 nor caspase-3 staining was prognostic. Lower tumour stage (II *vs* III) and lesser number of metastatic lymph nodes were significantly associated with more favourable DFS and OS rates (Table 1). Furthermore, tumours with better differentiation showed more favourable OS rates (Table 1). MMR status in tumours was not a significant prognostic variable (*n*=360) (Table 1).

In a multivariate analysis, EGFR intensity was analysed as a continuous variable and was independently associated with shorter DFS (HR: 1.18 (1.01, 1.38), *P*=0.042) (Table 3). EGFR intensity was also borderline significance for OS (HR: 1.15 (0.98, 1.34), *P*=0.086) in a model that included age, tumour stage, histological grade, and MMR status (Table 3). The analysis of EGFR dichotomised as strong (3+) intensity *vs* other intensities showed similar results for DFS (data not shown). Within this model, substituting the number of metastatic lymph nodes for tumour stage yielded similar results for EGFR intensity. Given the lack of association of phospho-EGFR and clinical outcome by univariate analysis, it was not studied multivariately. However, we examined the prognosis of the subset of tumours expressing both EGFR and phospho-EGFR compared with EGFR alone. No differences were found between these tumour subsets, thus suggesting that the EGFR activation status does not confer additional prognostic information.

## Discussion

Although EGFR expression has been studied in colorectal cancers (Lee *et al*, 2002; McKay *et al*, 2002; Spano *et al*, 2005), only limited and inconsistent data are available concerning the EGFR activation status in this malignancy. Furthermore, no studies have addressed the prognostic effect of phospho-EGFR in colon cancer patients. Accordingly, we analysed phospho-EGFR and EGFR expression in a large series of stage II and III colon cancers from completed 5-fluorouracil-based adjuvant therapy trials (O'Connell *et al*, 2006). Phospho-EGFR expression was detected in 157 of 388 (40%) colon cancers using an antibody that recognises the major EGFR autophosphorylation site at Tyr-1173. Tyr-1173 serves as a major binding site for Shc, an adaptor protein involved in signalling between EGFR and Ras (Okabayashi *et al*, 1994) and also as a binding site for phosphatases (Agazie and Hayman, 2003), that have an important role in modulating EGFR activity. Although the expression of phospho-EGFR was not associated with clinicopathological variables, both phospho-EGFR and EGFR were significantly associated with higher rates of cell proliferation, but not apoptosis, consistent with unopposed cell proliferation. The association of EGFR activation and expression with cell proliferation likely reflects downstream signalling, including Ras and ERK activation. ERK translocates to the nucleus and phosphorylates transcription factors that regulate genes controlling cell proliferation, including cyclin D1 (Cesana *et al*, 2006) that is overexpressed in 30–50% of colorectal carcinomas and is associated with a poor prognosis (Arber *et al*, 1996; McKay *et al*, 2000). Phospho-EGFR was not prognostic in our study which is concordant with the data obtained from non-small cell lung cancer and breast cancer patients, wherein no associations with survival were found (Cortas *et al*, 2007; Nieto *et al*, 2007). Although we studied a major EGFR phosphorylation site, it is possible that activation at other EGFR autophosphorylation sites may confer prognostic information.

We detected membranous EGFR expression using the EGFR pharmDx kit in 214 (59%) colon carcinomas. Potential mechanisms for EGFR expression include its transcriptional upregulation, decreased degradation, or gene amplification (Komuta *et al*, 1995; Shia *et al*, 2005). We found that strong EGFR intensity was significantly associated with higher tumour stage (III *vs* II) as compared with weaker EGFR intensity. EGFR protein expression was unrelated to tumour MMR status. This finding does not support a recent study that found elongation of a dinucleotide repeat in 55% of colon cancers with defective MMR, that was associated with significantly reduced EGFR mRNA levels (Baranovskaya *et al*, 2009). EGFR intensity as a continuous EGFR variable was significantly associated with shorter DFS and OS rates in a univariate analysis. EGFR was also a significant prognostic variable for DFS and OS when dichotomized as strong *vs* other intensity. In a recent study, strong (3+) EGFR staining was predictive of a high level of EGFR gene amplification (Hemmings *et al*, 2009), although conflicting reports exist (Milano *et al*, 2008). In a multivariate analysis, we found that EGFR intensity was independently associated with shorter DFS and was of borderline statistical significance for OS. Some (Lee *et al*, 2002; Resnick *et al*, 2004), but not other studies (McKay *et al*, 2002; Spano *et al*, 2005), have shown an adverse prognostic effect for EGFR expression in colorectal cancer patients. It is important to note that we used the U.S. FDA-approved EGFR pharmDx kit with its specific guidelines for EGFR scoring. Tumour stage, lymph node metastases, and histological grade were prognostic variables in our patient population. The lack of a survival advantage for tumours with defective MMR status may be related to the sample size and/or the observation that colon cancers with defective MMR do not respond to adjuvant 5-fluorouracil, whereas those with intact MMR receive a survival benefit from 5-fluorouracil treatment (Ribic *et al*, 2003).

Our findings are similar to a study in high-risk breast cancer patients where EGFR, but not phospho-EGFR, was an adverse prognostic factor (Nieto *et al*, 2007). This study utilised the phospho-EGFR Tyr 1086 antibody in breast cancers (Nieto *et al*, 2007) and reported a lack of association with EGFR, as was also found in our colorectal cancers and has been reported in other epithelial malignancies (Cunningham *et al*, 2005; Kong *et al*, 2006). However, excluding cases with phospho-EGFR Tyr-1173 but lacking EGFR staining resulted in a strong correlation between these two markers in our patient subset. We also examined whether the activation status of EGFR could alter the prognostic effect of EGFR. We found that tumours co-expressing phospho-EGFR and EGFR had a similar clinical outcome as those expressing EGFR alone. Therefore, our finding that EGFR, but not phospho-EGFR, is prognostic, suggests that the adverse effect on outcome is not mediated by EGFR activation at Tyr-1173, but by its cross-talk with other pathways.

Anti-EGFR therapies have been shown to inhibit EGFR phosphorylation and to reduce proliferation in human colon cancer cells and in tumour xenografts (Matar *et al*, 2004). Cell lines with ligand-induced EGFR phosphorylation, regardless of EGFR expression levels, show enhanced sensitivity to EGFR inhibitors (Lynch *et al*, 2004; Paez *et al*, 2004). Although IHC analysis of EGFR in colon cancers does not predict the efficacy of anti-EGFR antibody therapy (Ciardiello and Tortora, 2003; Chung *et al*, 2005; Van Cutsem *et al*, 2007), the potential predictive impact of phospho-EGFR expression for anti-EGFR antibodies in human colorectal cancers remains unknown. Given that the majority of patients in our adjuvant studies received 5-fluorouracil-based therapy, we were unable to address its predictive effect. In a limited number of metastatic colorectal carcinomas refractory to irinotecan, higher levels of phospho-EGFR expression were associated with better disease control in patients treated with cetuximab with or without irinotecan (Personeni *et al*, 2005). Furthermore, modulation of phospho-EGFR levels in skin biopsies during anti-EGFR therapy were shown to predict efficacy and may therefore, represent a potential surrogate biomarker (Agulnik *et al*, 2007). Such issues are highly relevant to locoregionally advanced colon cancers, as anti-EGFR antibodies are currently being studied in the adjuvant setting in this patient population.

In conclusion, phospho-EGFR expression was detected in 40% of stage II and III colon carcinomas. Both phospho-EGFR and EGFR overexpression were associated with tumour cell hyperproliferation; however, only EGFR was associated with adverse clinical outcome, thus suggesting that mechanisms other than EGFR activation may underlie its prognostic effect. These data are the first to analyze the prognostic effect of phospho-EGFR in human colon cancer patients, yet further study to examine its predictive utility for anti-EGFR therapy is warranted.

## Figures and Tables

**Table 1:** Univariate survival analysis for p-EGFR and EGFR staining intensity

**Parameter**	**Number of patients**	**5-yr DFS %**	**HR (95% CI)**	***P*-value^a^**	**5-yr OS %**	**HR (95% CI)**	***P*-value^a^**
*Age (years)*							
⩽65	216 (56%)	65.6	—	0.40	72.2	—	0.18
>65	172 (44%)	59.1	1.15 (0.83, 1.60)		68.6	1.24 (0.90, 1.72)	
							
*Gender*							
Female	185 (48%)	64.9	—	0.37	71.4	—	0.72
Male	203 (52%)	60.7	1.17 (0.84, 1.62)		69.9	1.06 (0.77, 1.46)	
							
*Tumour site*							
Distal	190 (49%)	61.9	—	0.60	71.1	—	0.78
Proximal	198 (51%)	63.5	0.92 (0.66, 1.27)		70.2	0.96 (0.69, 1.32)	
							
*Histological grade*							
Well/moderate	253 (65%)	65.1	—	0.10	74.3	—	0.038
Poor/undifferentiated	135 (35%)	58.3	1.32 (0.95, 1.85)		63.7	1.41 (1.02, 1.96)	
							
*Number of lymph nodes*							
0	77 (21%)	80.5	—	< 0.0001	87.0	—	< 0.0001
1–3	128 (35%)	66.7	1.78 (0.98, 3.24)		75.7	1.70 (0.95, 3.04)	
>3	158 (44%)	51.9	3.64 (2.05, 6.44)		60.1	3.70 (2.12, 6.46)	
							
*Tumour stage*							
Stage II	77 (20%)	80.5	—	0.0008	87.0	—	0.0008
Stage III	311 (80%)	58.3	2.43 (1.42, 4.15)		66.5	2.38 (1.41, 4.00)	
							
*MMR status*							
Intact MMR	327 (91%)	66.1	—	0.21	71.4	—	0.68
Defective MMR	33 (9%)	78.6	0.62 (0.29, 1.33)		84.6	0.88 (0.46, 1.67)	
							
*p-EGFR intensity*							
Absent (0)	231 (60%)	63.5			71.0		
Weak (1+)	133 (34%)	63.0	1.01 (0.77, 1.32)	0.95	68.4	1.08 (0.83, 1.40)	0.55
Moderate (2+)	24 (6%)	54.2			79.2		
							
*p-EGFR intensity*							
Absent (0)	231 (60%)	63.5	—	0.99	71.0	—	0.60
Present (1+, 2+)	157 (40%)	61.6	1.0 (0. 71, 1.40)		70.1	1.09 (0.78, 1.53)	
							
*EGFR intensity*							
Absent (0)	147 (41%)	73.3			78.0		
Weak (1+)	30 (8%)	66.7	1.21 (1.03, 1.41)	0.019	73.3	1.19 (1.02, 1.39)	0.028
Moderate (2+)	124 (34%)	66.5			72.4		
Strong (3+)	60 (17%)	54.7			59.7		
							
*EGFR intensity*							
Absent/weak/moderate (0, 1+, 2+)	301 (83%)	69.8	—	0.022	75.2	—	0.047
Strong (3+)	60 (17%)	54.7	1.65 (1.07, 2.53)		59.7	1.55 (1.00, 2.39)	

Abbreviations: CI, confidence interval; DFS, disease-free survival; EGFR, epidermal growth factor receptor; HR, hazard ratio; MMR, mismatch repair; OS, overall survival.

a Score *P*-value stratified by adjuvant study.

**Table 2:** Association of EGFR^a^ and phospho-EGFR expression with clinicopathologic variables

	**Phospho-EGFR intensity (*N*=388)**			**EGFR intensity (*N*=361)**		
**Characteristic**	**Absent (0) (*N*=231)**	**Weak/Moderate (1+, 2+) (*N*=157)**	***P*-value^b^**	**Other (0, 1+, 2+) (*N*=301)**	**Strong (3+) (*N*=60)**	***P*-value^b^**
*Age (years)*						
⩽65	123 (53%)	93 (59%)	0.24	179 (60%)	29 (48%)	0.11
>65	108 (47%)	64 (41%)		122 (40%)	31 (52%)	
						
*Gender*						
Female	107 (46%)	78 (50%)	0.52	142 (47%)	31 (52%)	0.52
Male	124 (54%)	79 (50%)		159 (53%)	29 (48%)	
						
*Tumour site*						
Proximal	111 (48%)	87 (55%)	0.15	134 (45%)	33 (55%)	0.14
Distal	120 (52%)	70 (45%)		167 (55%)	27 (45%)	
						
*Histological grade* c						
1, 2	146 (63%)	107 (68%)	0.32	217 (72%)	37 (62%)	0.11
3, 4	85 (37%)	50 (32%)		84 (28%)	23 (38%)	
						
*Lymph nodes*						
0	41 (18%)	36 (26%)	0.14	80 (27%)	8 (13%)	0.09
1–3	86 (38%)	42 (30%)		142 (47%)	32 (53%)	
>3	98 (44%)	60 (44%)		79 (26%)	20 (33%)	
						
*TNM*						
II	41 (18%)	36 (23%)	0.21	80 (27%)	8 (13%)	0.03
III	190 (82%)	121 (77%)		221 (73%)	52 (87%)	
						
*MMR status*						
Intact MMR	189 (91%)	116 (88%)	0.38	273 (91%)	54 (90%)	0.81
Defective MMR	19 (9%)	16 (12%)		27 (9%)	6 (10%)	
						
*Ki-67*						
Median (range)	15.7 (0.0–82.0)	26.2 (0.6–89.2)	0.015^d^,e	19.6 (0.0–89.2)	42.3 (1.7–85.8)	0.0002^d^
Mean (s.d.)	24.3 (21.7)	31.4 (22.8)		25.6 (21.9)	39.0 (24.9)	
						
*Caspase-3*						
Median (range)	0.0 (0.0–20.0)	0.0 (0.0–46.0)	0.24^d^,e	0.0 (0.0–35.0)	1.5 (0.0–46.0)	0.13^d^
Mean (s.d.)	2.1 (3.6)	3.9 (7.8)		2.7 (5.1)	3.9 (7.4)	
						
*Caspase-3 : Ki-67 ratio*						
Median (range)	0.0 (0.0–0.4)	0.0 (0.0–0.4)	0.54^d^,f	0.0 (0.0–1.4)	0.0 (0.0–0.3)	0.31^d^
Mean (s.d.)	0.0 (0.1)	0.0 (0.1)		0.0 (0.1)	0.0 (0.1)	

Abbreviations: EGFR, epidermal growth factor receptor; MMR, mismatch repair.

a Data are for membranous EGFR staining.

b Chi-square *P*-value.

c 1, 2 (well/moderate); 3, 4 (poor/undifferentiated).

d Wilcoxon's rank-sum *P*-value.

e 190 cases with p-EGFR and Ki67, caspase-3.

f 189 cases with p-EGFR and caspase-3 : Ki67 ratio.

**Table 3:** Multivariate analysis of EGFR intensity as a continuous variable

**Variable**	**Hazard ratio (95% CI)**	***P*-value^a^**
*Multivariate analysis for disease-free survival (N*=*360)*		
EGFR intensity (1 U increase)	1.18 (1.01, 1.38)	0.0415
Stage (III *vs* II)	1.99 (1.20, 3.30)	0.0040
Histological grade (3/4 *vs* 1/2)	1.44 (0.98, 2.13)	0.0683
Age (years) (> 65 *vs* ⩽65)	0.86 (0.59, 1.25)	0.4325
MMR status (defective *vs* intact)	0.53 (0.24, 1.15)	0.0771
		
*Multivariate analysis for overall survival (N*=*360)*		
EGFR intensity (1 U increase)	1.15 (0.98, 1.34)	0.0862
Stage (III *vs* II)	2.19 (1.31, 3.67)	0.0012
Histological grade (3/4 *vs* 1/2)	1.62 (1.11, 2.36)	0.0152
Age (years) (>65 *vs* ⩽65)	0.98 (0.68, 1.41)	0.9043
MMR status (defective *vs* intact)	0.69 (0.36, 1.34)	0.2540

Abbreviations: CI, confidence interval; EGFR, epidermal growth factor receptor; MMR, mismatch repair.

a Likelihood ratio *P*-value.

## References

[bib1] Agazie YM, Hayman MJ (2003) Molecular mechanism for a role of SHP2 in epidermal growth factor receptor signaling. Mol Cell Biol 23: 7875–7886 14560030 10.1128/MCB.23.21.7875-7886.2003 PMC207628

[bib2] Agulnik M, da Cunha Santos G, Hedley D, Nicklee T, Dos Reis PP, Ho J, Pond GR, Chen H, Chen S, Shyr Y, Winquist E, Soulieres D, Chen EX, Squire JA, Marrano P, Kamel-Reid S, Dancey J, Siu LL, Tsao MS (2007) Predictive and pharmacodynamic biomarker studies in tumor and skin tissue samples of patients with recurrent or metastatic squamous cell carcinoma of the head and neck treated with erlotinib. J Clin Oncol 25: 2184–2190 17538163 10.1200/JCO.2006.07.6554

[bib3] Allegra CJ, Parr AL, Wold LE, Mahoney MR, Sargent DJ, Johnston P, Klein P, Behan K, O'Connell MJ, Levitt R, Kugler JW, Tria Tirona M, Goldberg RM (2002) Investigation of the prognostic and predictive value of thymidylate synthase, p53, and Ki-67 in patients with locally advanced colon cancer. J Clin Oncol 20: 1735–1743 11919229 10.1200/JCO.2002.07.080

[bib4] Arber N, Hibshoosh H, Moss SF, Sutter T, Zhang Y, Begg M, Wang S, Weinstein IB, Holt PR (1996) Increased expression of cyclin D1 is an early event in multistage colorectal carcinogenesis. Gastroenterology 110: 669–674 8608874 10.1053/gast.1996.v110.pm8608874

[bib5] Baranovskaya S, Martin Y, Alonso S, Pisarchuk KL, Falchetti M, Dai Y, Khaldoyanidi S, Krajewski S, Novikova I, Sidorenko YS, Perucho M, Malkhosyan SR (2009) Down-regulation of epidermal growth factor receptor by selective expansion of a 5′-end regulatory dinucleotide repeat in colon cancer with microsatellite instability. Clin Cancer Res 15: 4531–4537 19584170 10.1158/1078-0432.CCR-08-1282 PMC2885604

[bib6] Boland CR, Thibodeau SN, Hamilton SR, Sidransky D, Eshleman JR, Burt RW, Meltzer SJ, Rodriguez-Bigas MA, Fodde R, Ranzani GN, Srivastava S (1998) A National Cancer Institute Workshop on Microsatellite Instability for cancer detection and familial predisposition: development of international criteria for the determination of microsatellite instability in colorectal cancer. Cancer Res 58: 5248–5257 9823339

[bib7] Buettner R, Mora LB, Jove R (2002) Activated STAT signaling in human tumors provides novel molecular targets for therapeutic intervention. Clin Cancer Res 8: 945–954 11948098

[bib8] Cesana GC, DeRaffele G, Cohen S, Moroziewicz D, Mitcham J, Stoutenburg J, Cheung K, Hesdorffer C, Kim-Schulze S, Kaufman HL (2006) Characterization of CD4+CD25+ regulatory T cells in patients treated with high-dose interleukin-2 for metastatic melanoma or renal cell carcinoma. J Clin Oncol 24: 1169–1177 16505437 10.1200/JCO.2005.03.6830

[bib9] Chattopadhyay A, Vecchi M, Ji Q, Mernaugh R, Carpenter G (1999) The role of individual SH2 domains in mediating association of phospholipase C-gamma1 with the activated EGF receptor. J Biol Chem 274: 26091–26097 10473558 10.1074/jbc.274.37.26091

[bib10] Chung KY, Shia J, Kemeny NE, Shah M, Schwartz GK, Tse A, Hamilton A, Pan D, Schrag D, Schwartz L, Klimstra DS, Fridman D, Kelsen DP, Saltz LB (2005) Cetuximab shows activity in colorectal cancer patients with tumors that do not express the epidermal growth factor receptor by immunohistochemistry. J Clin Oncol 23: 1803–1810 15677699 10.1200/JCO.2005.08.037

[bib11] Ciardiello F, Tortora G (2003) Epidermal growth factor receptor (EGFR) as a target in cancer therapy: understanding the role of receptor expression and other molecular determinants that could influence the response to anti-EGFR drugs. Eur J Cancer 39: 1348–1354 12826036 10.1016/s0959-8049(03)00235-1

[bib12] Compton C, Fenoglio-Preiser CM, Pettigrew N, Fielding LP (2000) American Joint Committee on Cancer Prognostic Factors Consensus Conference: Colorectal Working Group. Cancer 88: 1739–1757 10738234 10.1002/(sici)1097-0142(20000401)88:7<1739::aid-cncr30>3.0.co;2-t

[bib13] Cortas T, Eisenberg R, Fu P, Kern J, Patrick L, Dowlati A (2007) Activation state EGFR and STAT-3 as prognostic markers in resected non-small cell lung cancer. Lung Cancer 55: 349–355 17161498 10.1016/j.lungcan.2006.11.003

[bib14] Cox DR (1972) Regression models and life tables. JR Stat Soc 34: 187–202

[bib15] Cunningham MP, Essapen S, Thomas H, Green M, Lovell DP, Topham C, Marks C, Modjtahedi H (2005) Coexpression, prognostic significance and predictive value of EGFR, EGFRvIII and phosphorylated EGFR in colorectal cancer. Int J Oncol 27: 317–325 16010411

[bib16] Garrity MM, Burgart LJ, Mahoney MR, Windschitl HE, Salim M, Wiesenfeld M, Krook JE, Michalak JC, Goldberg RM, O'Connell MJ, Furth AF, Sargent DJ, Murphy LM, Hill E, Riehle DL, Meyers CH, Witzig TE (2004) Prognostic value of proliferation, apoptosis, defective DNA mismatch repair, and p53 overexpression in patients with resected Dukes' B2 or C colon cancer: a North Central Cancer Treatment Group Study. J Clin Oncol 22: 1572–1582 15117979 10.1200/JCO.2004.10.042

[bib17] Grambsch PMT, Terry M (1994) Proportional hazards tests and diagnostics based on weighted residuals. Biometrika 8: 515–526

[bib18] Gryfe R, Kim H, Hsieh ET, Aronson MD, Holowaty EJ, Bull SB, Redston M, Gallinger S (2000) Tumor microsatellite instability and clinical outcome in young patients with colorectal cancer. N Engl J Med 342: 69–77 10631274 10.1056/NEJM200001133420201

[bib19] Halling KC, French AJ, McDonnell SK, Burgart LJ, Schaid DJ, Peterson BJ, Moon-Tasson L, Mahoney MR, Sargent DJ, O'Connell MJ, Witzig TE, Farr Jr GH, Goldberg RM, Thibodeau SN (1999) Microsatellite instability and 8p allelic imbalance in stage B2 and C colorectal cancers. J Natl Cancer Inst 91: 1295–1303 10433618 10.1093/jnci/91.15.1295

[bib20] Hemmings C, Broomfield A, Bean E, Whitehead M, Yip D (2009) Immunohistochemical expression of EGFR in colorectal carcinoma correlates with high but not low level gene amplification, as demonstrated by CISH. Pathology 41: 356–360 19404848 10.1080/00313020902884477

[bib21] Jourdan F, Sebbagh N, Comperat E, Mourra N, Flahault A, Olschwang S, Duval A, Hamelin R, Flejou JF (2003) Tissue microarray technology: validation in colorectal carcinoma and analysis of p53, hMLH1, and hMSH2 immunohistochemical expression. Virchows Arch 443: 115–121 12802583 10.1007/s00428-003-0833-z

[bib22] Komuta K, Koji T, Izumi S, Matsumoto T, Kohara N, Motojima K, Kanematsu T, Nakane PK (1995) Expression of epidermal growth factor receptor messenger RNA in human colorectal carcinomas assessed by non-radioactive *in-situ* hybridization. Eur J Surg Oncol 21: 269–275 7781795 10.1016/s0748-7983(95)91426-9

[bib23] Kong A, Leboucher P, Leek R, Calleja V, Winter S, Harris A, Parker PJ, Larijani B (2006) Prognostic value of an activation state marker for epidermal growth factor receptor in tissue microarrays of head and neck cancer. Cancer Res 66: 2834–2843 16510606 10.1158/0008-5472.CAN-05-2994

[bib24] Lee JC, Wang ST, Chow NH, Yang HB (2002) Investigation of the prognostic value of coexpressed erbB family members for the survival of colorectal cancer patients after curative surgery. Eur J Cancer 38: 1065–1071 12008194 10.1016/s0959-8049(02)00004-7

[bib25] Lombardo CR, Consler TG, Kassel DB (1995) *In vitro* phosphorylation of the epidermal growth factor receptor autophosphorylation domain by c-src: identification of phosphorylation sites and c-src SH2 domain binding sites. Biochemistry 34: 16456–16466 8845374 10.1021/bi00050a029

[bib26] Lothe RA, Peltomaki P, Meling GI, Aaltonen LA, Nystrom-Lahti M, Pylkkanen L, Heimdal K, Andersen TI, Moller P, Rognum TO et al (1993) Genomic instability in colorectal cancer: relationship to clinicopathological variables and family history. Cancer Res 53: 5849–5852 8261392

[bib27] Lynch TJ, Bell DW, Sordella R, Gurubhagavatula S, Okimoto RA, Brannigan BW, Harris PL, Haserlat SM, Supko JG, Haluska FG, Louis DN, Christiani DC, Settleman J, Haber DA (2004) Activating mutations in the epidermal growth factor receptor underlying responsiveness of non-small-cell lung cancer to gefitinib. N Engl J Med 350: 2129–2139 15118073 10.1056/NEJMoa040938

[bib28] Matar P, Rojo F, Cassia R, Moreno-Bueno G, Di Cosimo S, Tabernero J, Guzman M, Rodriguez S, Arribas J, Palacios J, Baselga J (2004) Combined epidermal growth factor receptor targeting with the tyrosine kinase inhibitor gefitinib (ZD1839) and the monoclonal antibody cetuximab (IMC-C225): superiority over single-agent receptor targeting. Clin Cancer Res 10: 6487–6501 15475436 10.1158/1078-0432.CCR-04-0870

[bib29] McKay JA, Douglas JJ, Ross VG, Curran S, Murray GI, Cassidy J, McLeod HL (2000) Cyclin D1 protein expression and gene polymorphism in colorectal cancer. Aberdeen Colorectal Initiative. Int J Cancer 88: 77–81 10964085 10.1002/1097-0215(20001001)88:1<77::aid-ijc12>3.0.co;2-o

[bib30] McKay JA, Murray LJ, Curran S, Ross VG, Clark C, Murray GI, Cassidy J, McLeod HL (2002) Evaluation of the epidermal growth factor receptor (EGFR) in colorectal tumours and lymph node metastases. Eur J Cancer 38: 2258–2264 12441262 10.1016/s0959-8049(02)00234-4

[bib31] Milano G, Etienne-Grimaldi MC, Dahan L, Francoual M, Spano JP, Benchimol D, Chazal M, Letoublon C, Andre T, Gilly FN, Delpero JR, Formento JL (2008) Epidermal growth factor receptor (EGFR) status and K-Ras mutations in colorectal cancer. Ann Oncol 19: 2033–2038 18632722 10.1093/annonc/mdn416 PMC2733107

[bib32] Nieto Y, Nawaz F, Jones RB, Shpall EJ, Nawaz S (2007) Prognostic significance of overexpression and phosphorylation of epidermal growth factor receptor (EGFR) and the presence of truncated EGFRvIII in locoregionally advanced breast cancer. J Clin Oncol 25: 4405–4413 17906204 10.1200/JCO.2006.09.8822

[bib33] O'Connell MJ, Sargent DJ, Windschitl HE, Shepherd L, Mahoney MR, Krook JE, Rayson S, Morton RF, Rowland Jr KM, Kugler JW (2006) Randomized clinical trial of high-dose levamisole combined with 5-fluorouracil and leucovorin as surgical adjuvant therapy for high-risk colon cancer. Clin Colorectal Cancer 6: 133–139 16945169 10.3816/ccc.2006.n.030

[bib34] Okabayashi Y, Kido Y, Okutani T, Sugimoto Y, Sakaguchi K, Kasuga M (1994) Tyrosines 1148 and 1173 of activated human epidermal growth factor receptors are binding sites of Shc in intact cells. J Biol Chem 269: 18674–18678 8034616

[bib35] Paez JG, Janne PA, Lee JC, Tracy S, Greulich H, Gabriel S, Herman P, Kaye FJ, Lindeman N, Boggon TJ, Naoki K, Sasaki H, Fujii Y, Eck MJ, Sellers WR, Johnson BE, Meyerson M (2004) EGFR mutations in lung cancer: correlation with clinical response to gefitinib therapy. Science 304: 1497–1500 15118125 10.1126/science.1099314

[bib36] Parc YR, Halling KC, Wang L, Christensen ER, Cunningham JM, French AJ, Burgart LJ, Price-Troska TL, Roche PC, Thibodeau SN (2000) HMSH6 alterations in patients with microsatellite instability-low colorectal cancer. Cancer Res 60: 2225–2231 10786688

[bib37] Personeni N, Hendlisz A, Gallez J, Galdon MG, Larsimont D, Van Laethem J-L, Nagy N, Barette M, Paesmans M, Cardoso F, Bleiberg H (2005) Correlation between the response to cetuximab alone or in combination with irinotecan and the activated/phosphorylated epidermal growth factor receptor in metastatic colorectal cancer. Semin Oncol 32: 59–62 10.1053/j.seminoncol.2005.04.029 16399434

[bib38] Piazzi G, Paterini P, Ceccarelli C, Pantaleo MA, Biasco G (2006) Molecular determination of epidermal growth factor receptor in normal and neoplastic colorectal mucosa. Br J Cancer 95: 1525–1528 17088913 10.1038/sj.bjc.6603441 PMC2360732

[bib39] Radinsky R, Risin S, Fan D, Dong Z, Bielenberg D, Bucana CD, Fidler IJ (1995) Level and function of epidermal growth factor receptor predict the metastatic potential of human colon carcinoma cells. Clin Cancer Res 1: 19–31 9815883

[bib40] Resnick MB, Routhier J, Konkin T, Sabo E, Pricolo VE (2004) Epidermal growth factor receptor, c-MET, beta-catenin, and p53 expression as prognostic indicators in stage II colon cancer: a tissue microarray study. Clin Cancer Res 10: 3069–3075 15131045 10.1158/1078-0432.ccr-03-0462

[bib41] Ribic CM, Sargent DJ, Moore MJ, Thibodeau SN, French AJ, Goldberg RM, Hamilton SR, Laurent-Puig P, Gryfe R, Shepherd LE, Tu D, Redston M, Gallinger S (2003) Tumor microsatellite-instability status as a predictor of benefit from fluorouracil-based adjuvant chemotherapy for colon cancer. N Engl J Med 349: 247–257 12867608 10.1056/NEJMoa022289 PMC3584639

[bib42] Roberts RB, Min L, Washington MK, Olsen SJ, Settle SH, Coffey RJ, Threadgill DW (2002) Importance of epidermal growth factor receptor signaling in establishment of adenomas and maintenance of carcinomas during intestinal tumorigenesis. Proc Natl Acad Sci USA 99: 1521–1526 11818567 10.1073/pnas.032678499 PMC122223

[bib43] Shia J, Klimstra DS, Li AR, Qin J, Saltz L, Teruya-Feldstein J, Akram M, Chung KY, Yao D, Paty PB, Gerald W, Chen B (2005) Epidermal growth factor receptor expression and gene amplification in colorectal carcinoma: an immunohistochemical and chromogenic *in situ* hybridization study. Mod Pathol 18: 1350–1356 15832190 10.1038/modpathol.3800417

[bib44] Sinicrope FA, Rego RL, Garrity-Park MM, Foster NR, Sargent DJ, Goldberg RM, Wiesenfeld M, Witzig TE, Thibodeau SN, Burgart LJ (2007) Alterations in cell proliferation and apoptosis in colon cancers with microsatellite instability. Int J Cancer 120: 1232–1238 17187355 10.1002/ijc.22429

[bib45] Sinicrope FA, Rego RL, Halling KC, Foster N, Sargent DJ, La Plant B, French AJ, Laurie JA, Goldberg RM, Thibodeau SN, Witzig TE (2006a) Prognostic impact of microsatellite instability and DNA ploidy in human colon carcinoma patients. Gastroenterology 131: 729–737 16952542 10.1053/j.gastro.2006.06.005

[bib46] Sinicrope FA, Rego RL, Halling KC, Foster NR, Sargent DJ, La Plant B, French AJ, Allegra CJ, Laurie JA, Goldberg RM, Witzig TE, Thibodeau SN (2006b) Thymidylate synthase expression in colon carcinomas with microsatellite instability. Clin Cancer Res 12: 2738–2744 16675565 10.1158/1078-0432.CCR-06-0178

[bib47] Sinicrope FA, Sargent DJ (2009) Clinical implications of microsatellite instability in sporadic colon cancers. Curr Opin Oncol 21: 369–373 19444104 10.1097/CCO.0b013e32832c94bd PMC3761884

[bib48] Spano JP, Lagorce C, Atlan D, Milano G, Domont J, Benamouzig R, Attar A, Benichou J, Martin A, Morere JF, Raphael M, Penault-Llorca F, Breau JL, Fagard R, Khayat D, Wind P (2005) Impact of EGFR expression on colorectal cancer patient prognosis and survival. Ann Oncol 16: 102–108 15598946 10.1093/annonc/mdi006

[bib49] Thibodeau SN, Bren G, Schaid D (1993) Microsatellite instability in cancer of the proximal colon. Science 260: 816–819 8484122 10.1126/science.8484122

[bib50] Thibodeau SN, French AJ, Cunningham JM, Tester D, Burgart LJ, Roche PC, McDonnell SK, Schaid DJ, Vockley CW, Michels VV, Farr Jr GH, O'Connell MJ (1998) Microsatellite instability in colorectal cancer: different mutator phenotypes and the principal involvement of hMLH1. Cancer Res 58: 1713–1718 9563488

[bib51] Van Cutsem E, Peeters M, Siena S, Humblet Y, Hendlisz A, Neyns B, Canon JL, Van Laethem JL, Maurel J, Richardson G, Wolf M, Amado RG (2007) Open-label phase III trial of panitumumab plus best supportive care compared with best supportive care alone in patients with chemotherapy-refractory metastatic colorectal cancer. J Clin Oncol 25: 1658–1664 17470858 10.1200/JCO.2006.08.1620

[bib52] Wang W-S, Chen P-M, Chiou T-J, Liu J-H, Lin J-K, Lin T-C, Wang H-S, Su Y (2007) Epidermal growth factor receptor R497K polymorphism is a favorable prognostic factor for patients with colorectal carcinoma. Clin Cancer Res 13: 3597–3604 17575224 10.1158/1078-0432.CCR-06-2601

[bib53] Yarden Y, Sliwkowski MX (2001) Untangling the ErbB signalling network. Nat Rev Mol Cell Biol 2: 127–137 11252954 10.1038/35052073

